# Ligature‐associated bacterial profiles are linked to type 2 diabetes mellitus in a rat model and influenced by antibody treatment against TNF‐α or RAGE

**DOI:** 10.1002/cre2.54

**Published:** 2017-02-27

**Authors:** M.B. Grauballe, D. Belstrøm, J.A. Østergaard, B.J. Paster, S. Schou, A. Flyvbjerg, P. Holmstrup

**Affiliations:** ^1^ Section for Periodontology, Department of Dentistry, Faculty of Health Aarhus University Aarhus C Denmark; ^2^ Section for Periodontology, Microbiology and Community Dentistry, Department of Odontology, Faculty of Health and Medical Sciences University of Copenhagen Copenhagen N Denmark; ^3^ The Medical Research Laboratories, Department of Clinical Medicine, Faculty of Health, Aarhus University and Department of Endocrinology and Internal Medicine Aarhus University Hospital Aarhus C Denmark; ^4^ Danish Diabetes Academy Odense Denmark; ^5^ The Forsyth Institute Department of Microbiology Cambridge, MA USA; ^6^ Department of Oral Medicine, Infection & Immunity Harvard School of Dental Medicine Boston, MA USA; ^7^ Section for Oral Surgery and Oral Pathology, Department of Odontology, Faculty of Health and Medical Sciences University of Copenhagen Copenhagen N Denmark; ^8^ Department of Endocrinology and Internal Medicine Aarhus University Hospital Denmark

**Keywords:** anti‐RAGE, anti‐TNF‐α, diabetes mellitus, diabetes type 2, Periodontal disease, Periodontitis, stages

## Abstract

There is a bidirectional relationship between periodontal disease (PD) and type 2 diabetes mellitus (T2D). T2D may lead to ecological perturbations in the oral environment, which may facilitate an altered microbiota. However, previous studies have been inconclusive in determining the effect of T2D on oral bacterial profiles. Therefore, we aimed to evaluate the influence of T2D on the ligature‐associated bacterial profile in a diabetic rat model with PD and investigated the impact of blocking inflammatory pathways with antibodies targeting either Tumor Necrosis Factor α (TNF‐α) or the receptor of advanced glycation end‐products (RAGE). A total of 62 Zucker obese rats (45 T2D) and 17 lean (non‐T2D) were divided into 4 treatment groups; lean with PD, obese with PD, obese with PD and anti‐TNF‐α treatment, and obese with PD with anti‐RAGE treatment. Periodontal disease was ligature induced. Ligature‐associated bacterial profiles were analyzed using Human Oral Microbe Identification Microarray (HOMIM). Ligature‐associated bacterial profiles differed between lean and obese rats. Furthermore, treatment with antibodies against TNF‐α or RAGE had an impact on subgingival bacterial profiles. T2D phenotypes are associated with different ligature‐associated bacterial profiles and influenced by treatment with antibodies against TNF‐α or RAGE.

## 1 INTRODUCTION

Periodontal disease (PD) and type 2 diabetes mellitus (T2D) are highly prevalent chronic inflammatory diseases (Taylor, 2001). Biofilm‐mediated periodontal inflammation seems to be critical in periodontal degradation (Gemmell, Yamazaki, & Seymour, 2002), as bacterial alterations of the biofilm may be associated with an increased risk of progressive disease (Darveau, 2010). The hyperglycemic state in T2D increases the formation of advanced glycation end‐products (AGE). AGE alter the immune response in T2D with up‐regulation of pro‐inflammatory cytokines, e.g., Tumor Necrosis Factor‐α (TNF‐α) (Ramasamy, Yan, & Schmidt, 2012), which in turn is believed to influence the progression of PD (Lalla & Papapanou, 2011). Furthermore, T2D affects the oral environment with higher glucose levels and pro‐inflammatory mediators in the gingival crevicular fluid, which have been regarded key mechanisms for modifying the microbiota in T2D patients (Engebretson et al., 2004; Ficara, Levin, Grower, & Kramer, 1975). Recent studies have demonstrated different bacterial profiles of subgingival plaque in T2D patients with chronic PD as compared to PD patients without T2D (Casarin et al., 2013; Zhou et al., 2013). Furthermore, in the Oral Infections, Glucose Intolerance, and Insulin Resistance Study, increasing colonization levels of periodontal pathogens were observed with increasing prevalence of prediabetes (Demmer et al., 2015). However, other studies have been not demonstrated such changes of the microbiota (Taylor, Preshaw, & Lalla, 2013).

Due to heterogeneity of microbial and host factors in human subjects, it may be difficult to attribute specific bacterial associations to disease status. Consequently, standardized animal models with controlled diet, housing and microbial sampling may be preferable for these studies (Kaye, 2012), which is why rat models with ligature‐induced PD have been used frequently. Animal models with both T2D and PD have increased inflammatory cytokine responses, elevated oxidative stress, raised AGE levels, altered glucose metabolism, and increased severity of PD (Pontes Andersen, Flyvbjerg, Buschard, & Holmstrup, 2007). In addition to being a local irritant, the ligature serves as a reservoir for bacteria, which mediates periodontal tissue destruction (Bjornsson et al., 2003). Male Zucker obese rats (ZOR) display a mutation in their leptin receptors and will therefore develop obesity, hyperlipidemia and mild hyperglycemia. Thus, Zucker obese rats are widely used in T2D studies (Chen & Wang, 2005).

The development of high throughput molecular methods, primarily based on analysis of the bacterial 16S rRNA gene, has enabled comprehensive analysis of bacterial community changes in relation to health and disease (Paster & Dewhirst, 2009). Thus, in the present study, we used the Human Oral Microbe Identification Microarray (HOMIM) for analysis of ligature‐associated bacterial profiles in a rat model with T2D and PD. The aim of the study was to compare ligature‐associated bacterial profiles in ZOR (T2D) to that of lean non‐T2D Zucker rats. Furthermore, we compared ligature‐associated bacterial profiles in ZOR treated with Etanercept (ETN), a TNF‐α blocking antibody, or an antibody (ARA) blocking the receptor of AGE (RAGE). We hypothesized that oral bacterial profiles in T2D rats differ from that of non‐diabetic rats with impact of antibodies targeting diabetes‐associated pro‐inflammatory cytokines and antibodies targeting RAGE.

## 2 MATERIALS AND METHODS

The present study was undertaken as part of a group of studies on experimental PD and T2D in rats, the additional results are presented elsewhere (Grauballe, Ostergaard, Schou, Flyvbjerg, & Holmstrup, 2015; Grauballe, Ostergaard, Schou, Flyvbjerg, & Holmstrup, 2016).

### 2.1 Animals

The present study was undertaken as part of a group of studies on experimental PD and T2D in rats, and the additional results are presented elsewhere (17;18).

Four week old male ZORs (HsdHlr:ZUCKER‐*Lepr* ^*fa/fa*^ n=45) and lean controls (ZUCKER‐*Lepr* ^*fa/+*^n= 17) (Harlan Laboratories, Livermore, USA) were included. Animals were housed in pairs and kept at a constant temperature (21°±1°C). Cages were placed in a room with an artificial light cycle (dark 7.00 p.m. to 7.00 a.m.) and a humidity of 55±5%.

To avoid spontaneous PD (Bjornsson et al., 2003), the animals were bred on Teklad 7089 Diamond Soft Bedding (Harlan Laboratories, Livermore, CA, USA). The rat pups were placed on the special bedding before they were 15 days old, with free access to food (Purina #5008, LabDiet, St. Louis, MO, USA) and water. The animals had one week of acclimatization before start of the experiments.

A license to perform the study was obtained from the Ethical Committee for Animal Research, Department of Justice, Copenhagen, Denmark (J.no. 2012‐15‐2934‐00455).

### 2.2 Experimental design

PD was induced by placing ligatures around the cervix of 2^nd^ upper molars under general anaesthesia (Dormicum, Vetapharma, Leeds, UK and Hypnorm, Roche, Basel, Switzerland, 0.20 ml/100 g body weight). Before PD was induced, all rats were examined to exclude rats with pre‐existing PD (defined by probing depths >0.5 mm) (Bjornsson et al., 2003). Each week, PD induction was checked under general anesthesia and loose or lost ligatures were replaced as previously described (17; 18).

The study was designed with the following groups:

1. Lean, normoglycemic control rats (non‐T2D + PD); LPD (n=17): control animals with PD. Subcutaneous saline injections 3 times a week as placebo throughout the study.

The obese rats were randomly divided into three groups:

1. Obese (T2D + PD); Obese animals with PD. OPD (n=15): Subcutaneous saline injections 3 times a week as placebo throughout the study.
2. Obese (T2D + PD) + ETN (anti‐TNF‐α); Obese animals with PD. OPDE (n=15): Subcutaneous injections of 0.5 ml of 0.78 mg/ml ETN (Wyeth, Glostrup, Denmark) 3 times a week throughout the study.
3. Obese (T2D) + PD) + ARA (anti‐RAGE); Obese animals with PD. OPDAR (n=15): Intraperitoneal injections of 0.8 ml of 1.25 g/l ARA 3 times a week throughout the study.

At baseline an Oral Glucose Tolerance Test (OGTT) was performed in all groups. The following day antibody treatment was initiated. One week later, PD was induced under general anaesthesia. Body weight and blood glucose levels were recorded at baseline and each week.

At week 4, food and water consumption and urine and feces production were measured in 12 randomly selected rats from each group, which were placed in metabolic cages (TECNIPLAST, Buguggiate, Italy) for 20 h. At week 5, final OGTTs were performed and at the end of week 5, all rats were euthanized under general anaesthesia with pentobarbital (Glostrup Apotek, Glostrup, Denmark) (100 mg/kg intra‐peritoneal) (Grauballe et al., 2015). Rats were decapitated and ligatures were removed with sterile forceps and both ligatures from each rat were put into one sterile tube containing MagNa pure bacteria lysis buffer (Roche, Mannheim, Germany) and stored at −80°C until DNA isolation.

### 2.3 DNA isolation and HOMIM analysis

DNA isolation from subgingival ligatures was performed according to manufacturer's specifications, using the protocol *Pathogen Universal 200* (Roche, Mannheim, Germany). Extracted DNA was stored at ‐80°C until analyzed by the Human Oral Microbe Identification Microarray (HOMIM). The laboratory procedures of HOMIM have been presented in detail (Colombo et al., 2009). Briefly, HOMIM is a molecular method using two consecutive polymerase chain reactions (PCR) and a subsequent DNA‐DNA hybridization for identification of around 300 human oral bacterial species. Thus, fluorescence‐labeled single stranded PCR products are captured by complementary single stranded oligonucleotide‐probes (18‐20 bases long), printed on a customized array, as the probes target highly variable areas of the phylogenetically informative 16S ribosomal RNA gene. Initially, the quality of the PCR products were assured using an agarose gel with comparison of positive and negative control samples. In addition, the quantity and quality of DNA was measured by using a NanoDrop 8000 Spectrophotometer (Thermo Scientific, Waltham, Massachusetts, USA). Thus, only samples with a DNA content >10 μg/l and a 260/280 ratio of >1.8 were further analyzed. Data were collected using an Axon 4000B scanner, and crude data analysis was performed with Genepix 6 software (Molecular Devices, Sunnyvale, CA, USA). Mean fluorescence intensity was calculated for each probe and normalized by values from positive universal probes, negative controls and buffer spots. A semi‐quantitative HOMIM‐value from 1 to 5 was calculated for each probe. In this study the newest version of HOMIM (version 5) was used. Further analysis and generation of microbial profiles were carried out using the HOMIM online tool (http://bioinformatics.forsyth.org/homim/ (accessed on 15^th^ of October, 2015)).

### 2.4 Statistical analysis

All data were checked for normality using QQ‐plots. Data from metabolic cages are presented as mean ± SD, and groups are compared using one‐way ANOVA followed by Tukeys multiple comparisons test. For these analyses a p‐value < 0.05 were considered as statistically significant. Bacterial profiles were compared between groups at probe level, using information of frequency (mean presence) and levels (mean HOMIM‐value) from each probe included on the HOMIM microarray as endpoints. These analyses were performed with Mann‐Whitney test and Kruskal‐Wallis test when two groups and three groups were compared respectively, and results were adjusted according to Benjamini‐Hochberg's correction for multiple testing (Hochberg & Benjamini, 1990). For comparisons at probe level, only an adjusted p‐value < 0.01 was considered statistically significant to further minimize the risk of type 1 errors. Principal component analysis was used for visualizing differences in bacterial community profiles. All analyses were performed using the statistical software packages of Graphpad Prism 5 (San Diego, CA, USA) and MeV 4_8_1 (Saeed et al., 2006).

## 3 RESULTS

All data concerning anti‐TNF‐α (Grauballe et al., 2015) and anti‐RAGE (Grauballe et al., 2016) treatment on periodontal and glycemic status has been presented elsewhere. In brief, periodontal bone support in the obese groups (T2D) was decreased except for the OPDE group which could not be separated from the lean LPD group (non T2D). (Grauballe et al., 2015).Treatment with anti‐RAGE had no influence on periodontal support, compared to that of the control group (Grauballe et al., 2016). Furthermore, anti‐TNF‐α treatment significantly improved the insulin resistance (Grauballe et al., 2015), and anti‐RAGE significantly improved both glucose tolerance and insulin resistance compared to the control group (Grauballe et al., 2016).

### 3.1 Animals

**Figure 1:**
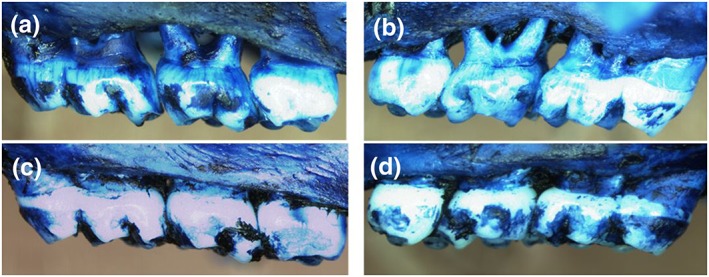
Photographs of ligature‐induced PD. (a) LPD (lean with periodontal disease), (b) OPD (obese with periodontal disease), (c) LC (lean without periodontal disease), (d) OC (obese without periodontal disease)

A total of 10 rats died during the study period due to either general anesthesia or failed glucose gavage into the trachea. Thus a total of 52 animals completed the entire study; from which 51 ligatures were successfully collected (One set of ligatures from one rat in the OPDE group was lost during collection). Morphometric presentation of animals is shown in Figure 1a‐d. In addition, 47 recordings from metabolic cages were performed (one recording was discharged due to wrong assembly of the cage). Data from metabolic cages (production of urine and stool, consumption of food and water and weight gain) are presented in Table 1. In brief, only inconsiderable differences were observed on all parameters recorded. Urine production was significantly lower in the LPD group compared to OPD group (P<0.01), but no difference could be detected in the obese (T2D) groups (OPD, OPDE and OPDAR).

**Table 1:** Means and standard deviation of stool, urine, food consumption, water consumption, and weight gain during placement for 20‐hours in metabolic cages

Group	LPD	OPD	OPDE	OPDAR
Stool (g)				
Mean	18.09	21.47	21.09	18.58
Std. Deviation	3.26	3.48	2.91	3.91
Urine (ml)				
Mean	8.23**	13.96**	11.73	12.48
Std. Deviation	1.64	6.43	4.28	2.41
Food consumption (g)
Mean	30.8	35.6	34.47	32.52
Std. Deviation	5.37	2.75	4.43	7.38
Water consumption (ml)				
Mean	24.08	25.12	24.73	27.46
Std. Deviation	3.46	6.76	5.07	4.95
Weight gain (g)				
Mean	4.16	3.14	3.26	4.29
Std. Deviation	3.03	5.91	7.32	3.43

LPD (Lean+periodontal disease) *n* = 12, OPD (Obese+periodontal disease) *n* = 11, OPDE (obese+periodontal disease+Etanercept treatment) *n* = 12, OPDAR (obese+periodontal disease+RAGE antibody treatment) *n* = 12.

** P < 0.01

### 3.2 General findings from HOMIM

From a total of 51 samples, positive identification of the target of 50 probes was recorded (25 probes recognizing a bacterial taxon and 25 probes recognizing a bacterial cluster), meaning that 13% of the 383 targets identified by the HOMIM technology were recorded in this cohort of samples. The mean number of targets identified in the total cohort was 16, with a range from 8 to 27. Seven different phyla were identified (Firmicutes, Proteobacteria, Enterobacteria, Actinobacteria, Bacteroidetes, Fusobacteria and Spirochetes). Firmicutes was the predominant phylum identified accounting for 60% of the total probe signal recorded, and the predominant genus was *Streptococcus* accounting for 34% of the total probe signal. No differences in mean number of probes identified or phylogenic distribution between the four subgroups were observed. A complete list of probes present on the HOMIM microarray is presented in Supplementary material 1, and information of frequency and mean‐HOMIM values in the total amount of samples and in each of the four groups of probes identified, is listed in Supplementary material 2.

### 3.3 The ligature‐associated microbial community profile differs between LPD and OPD

**Figure 2:**
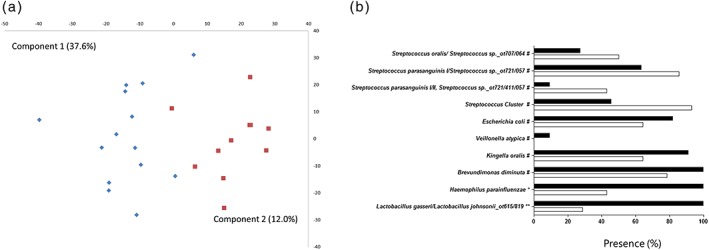
Ligature‐associated bacterial profiles in LDP (lean) and OPD (T2D) groups. (a) Principal component analysis displaying component 1 (x‐axis) and component 2 (y‐axis) accountable of 49.6 % of the total mathematical variation of the dataset. Blue: LPD, Red: OPD. (b) Presence of predominant taxon/cluster in % of total samples. White bars: LPD. Black bars: OPD. *: adjusted p‐value<0.05, **: adjusted p‐value<0.01, #: adjusted p‐value>0.05

Principal component analysis showed different bacterial community profiles between OPD (n=11) and LPD (n=14). There was an almost complete separate clustering of samples from the two groups based on the principal component analysis of the dataset accounting for 37.6% of the variation in the cohort analyzed (Figure 2a). Comparison at taxon/cluster level showed that the bacterial cluster *Lactobacillus gasseri/Lactobacillus johnsonii* HOT 615/819 was significantly more frequently identified in samples from OPD (adjusted p‐value<0.01). In addition, *Haemophilus parainfluenzae* was also identified more frequently in the OPD group (adjusted p‐value<0.05). Furthermore, several bacterial taxa and clusters were recorded with differences in frequency between the two groups, although these observations were non‐significant when adjusted for multiple dependent assumptions. The 10 most decisive probes (recognizing 6 bacterial taxa and 4 bacterial clusters) are presented in (Figure 2b).

### 3.4 Major differences in bacterial community profiles between OPD and antibody treated ZOR (OPDE and OPDAR)

**Figure 3:**
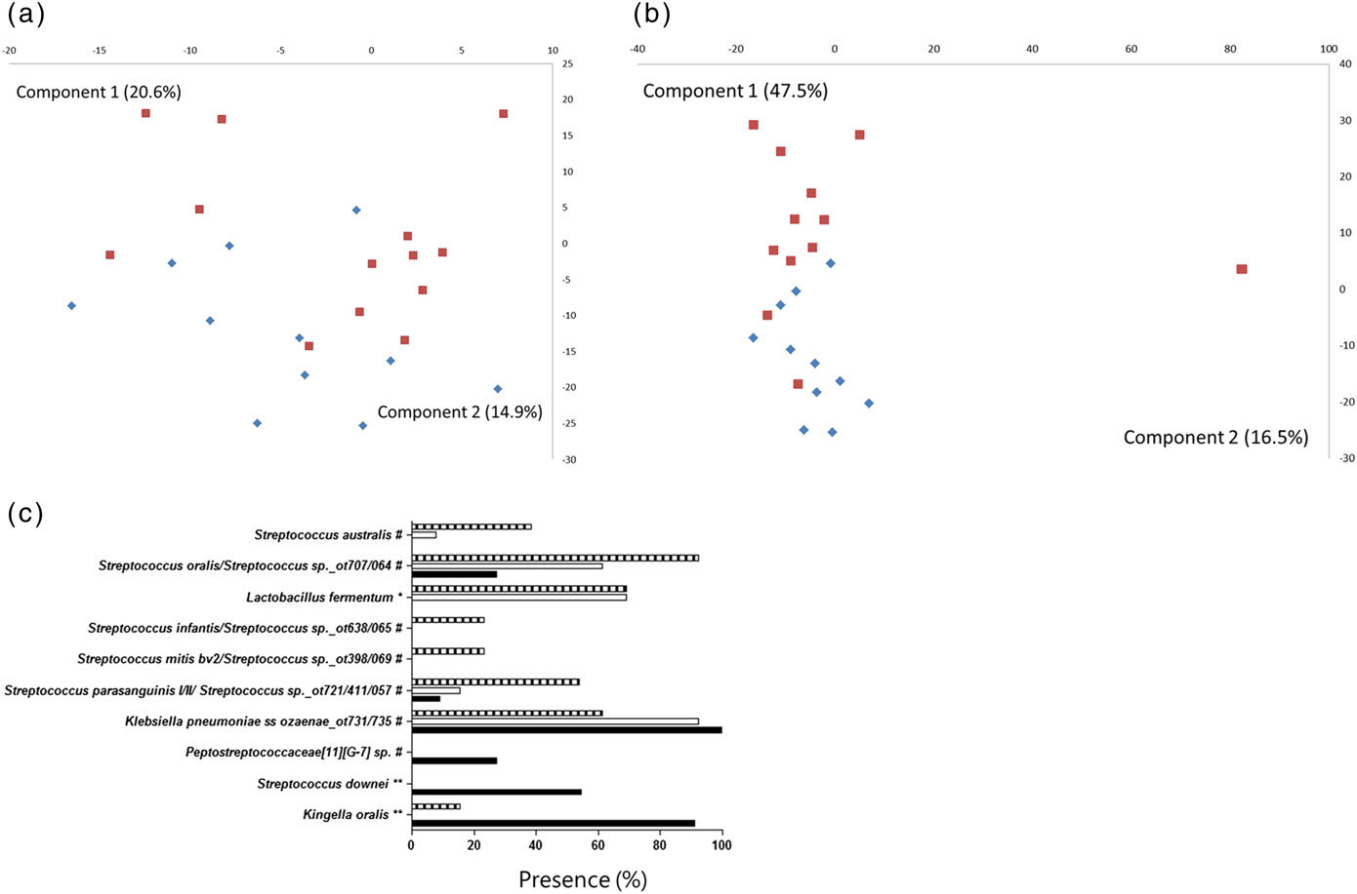
Ligature‐associated bacterial profiles in the OPD (T2D), OPDE (anti‐TNF‐α) and OPDAR (anti‐RAGE) groups. (a) Principal component analysis is visualized by component 1 (x‐axis) and component 2 (y‐axis) accountable of 35.5 % of the total mathematical variation of the dataset. Blue: OPD, Red: OPDE. (b) Principal component analysis showing component 1 (x‐axis) and component 2 (y‐axis) accountable of 64.0 % of the total mathematical variation of the dataset. Blue: OPD, Red: OPDAR. (c) Presence of predominant taxon/cluster in % of total samples. Black bars: OPD, White bars: OPDE, Dotted bars: OPDAR. *: adjusted p‐value<0.05, **: adjusted p‐value<0.01, #: adjusted p‐value>0.05

The bacterial community profile of OPD (n=11) was compared to the bacterial community profiles of OPDE (n=13) and OPDAR (n=13), respectively. As seen in (Figure 3a‐b) the samples from OPD clustered almost completely separate from the antibody treated diabetes groups based on component 2 accountable for 14.9% and 16.5% of the mathematical variation in the two datasets respectively. The two bacterial taxa *Kingella oralis* and *Streptococcus downei* were identified significantly more often in the OPD group than in the two antibody treated groups (adjusted p‐value<0.01). In addition, the species *Lactobacillus fermentum* was identified more often in the OPDE and OPDAR group than in the untreated group (adjusted p‐value <0.05). Furthermore, differences at taxon/cluster level were observed between groups, although insignificant. The 10 most decisive probes (recognizing 5 bacterial taxa and 5 bacterial clusters) based on differences in frequency are presented in (Figure 3c).

## 4 DISCUSSION

The aim of the present investigation was to compare ligature‐associated bacterial profiles in rats with T2D to that of lean rats. The main finding was that ligature‐associated bacterial profiles were linked to T2D, and that treatment with an antibody targeting either TNF‐α or RAGE had an impact on bacterial profiles. To the best of our knowledge, this is the first study to investigate if antibodies targeting pro‐inflammatory cytokines have an impact on the oral microbiota in a rat model with PD and T2D.

Some limitations apply to the results presented in this study. Experimental PD in rats is based upon subgingival placement of ligatures that facilitate accumulation of bacteria, which in turn initiate periodontal inflammation (Graves, Fine, Teng, Van Dyke, & Hajishengallis, 2008; Pontes Andersen et al., 2007). Thus, subgingival placement of the ligature increases biofilm formation, and a direct comparison between ligature‐induced PD in rats and naturally occurring PD in humans is not possible (Graves et al., 2008). It is, however, interesting that while trauma from the ligature obviously play a role in periodontal disease progression in the PD rat model, a study in germfree rats showed that no bone loss occurred in the absence of biofilm accumulation (Rovin, Costich, & Gordon, 1966). Thus, progressive bone loss in the ligature‐induced PD model in rats seems to be dependent on bacterial accumulation and subsequent colonization, which suggests that the model is suitable for studies on subgingival bacterial profiles in PD.

Since sterile silk 4.0 ligatures were used to induce PD, disparities in frequency of ligatures being replaced and lost, and variation of subgingival placement of ligatures may account for variation in PD and biofilm composition in each group. However, in this study the same frequency of lost ligatures was observed in each group (data not shown).

Moreover, bacterial identifications in this study are based on data generated by HOMIM, which is a molecular technique developed for analysis of human oral bacterial species. Therefore, the organisms identified must be interpreted with caution and consequently the terminology “‐like species” is used as in previous reports (Duarte, Tezolin, Figueiredo, Feres, & Bastos, 2010; Rober, Quirynen, Haffajee, Schepers, & Teughels, 2008). Furthermore, bacterial species with genotypes different from the 300 present in HOMIM are not likely recognized, and bacteria recognized on the microarray may show phenotypes different from human bacteria (Fournier et al., 2001).

Obviously, contemporary molecular methods developed for analysis of human bacteria might not be ideal for analysis of rat‐associated bacteria. However, since few to none methods are developed specifically for analysis of rat‐associated bacteria, this might be the most feasible and cost‐effective choice, even though the composition of the oral biofilm in rats with experimental PD is likely different from that of humans with PD. It is therefore noteworthy, that a study performed on Wistar rats using DNA‐DNA hybridization, reported positive identification of 25 out of 40 pre‐selected human bacterial species, including some bacteria from the red complex (Duarte et al., 2010). Interestingly, 6 bacterial species identified in the aforementioned investigation were also identified in this study. However, in the present study periopathogens from the red complex (Socransky, Haffajee, Cugini, Smith, & Kent, 1998) were not detected, which is in line with another study in a diabetes type 1 rat model (Claudino et al., 2012).

Notably, *Veillonella parvula*‐like species were found in all samples analyzed in this study (Supplementary material 2). In humans, *V. parvula* is known to be an early colonizer establishing an environment for late colonizers including the human periodontal pathogen *Fusobacterium periodonticum. F. periodonticum‐like* species were detected in high proportions in this study. Studies have suggested that colonization by *Fusobacterium* spp. is essential for colonization of periodontal pathogens (Kolenbrander et al., 2006). It is therefore noteworthy that culture‐based techniques have been used to demonstrate that high proportions of *Fusobacterium*‐like species is associated with the presence of a matured biofilm in rats (Isogai, Isogai, Sawada, Kaneko, & Ito, 1985). Essentially, this reinforces the assumption that subgingival ligatures initiate periodontal disease in rats by acting as a scaffold for biofilm formation and maturation. On another note, *Rothia* spp. has been reported to constitute a predominant part of the indigenous rat oral microbiome (Manrique et al., 2013), which is conflicting with data from the present study, in which *Rothia* spp. were not detected. This finding suggests that *Rothia* spp. might associate with oral health in rats. However, as different samples (supragingival vs. subgingival) were collected and various molecular methods (pyrosequencing vs. HOMIM) employed for bacterial analysis in the two investigations, future studies are warranted.

Ideally, analysis of T2D‐associated bacterial alterations should be carried out in humans. However, the main limitation with this approach is that the complex interplay between PD and T2D can make it difficult to distinguish whether any observed differences in subgingival bacterial profiles are due to PD status or the result of diabetic state (Taylor et al., 2013). On the other hand, the main advantage of using animal models is that selected aspects of a complex interaction between diseases may be studied in a controlled manner. It is therefore interesting that while comparable levels of PD were identified, highly significant differences in the diabetic state was present in the lean (LPD) group compared with the T2D (OPD) group (17;18). Thus, differences in ligature‐associated bacterial profiles in the LPD and OPD group (Figure 2a‐b) were most probably a result of the increased inflammatory state in the OPD group. In line, these findings were further reinforced as selective treatment with anti‐TNF (OPDE group) or anti‐RAGE (OPDAR group) antibodies positively influenced the OPDE group and OPDAR group towards a healthier diabetic phenotype (17;18), which in turn displayed ligature‐associated bacterial profiles different from that of the OPD group (Figure 3a‐c). Notably, these findings suggest that improvement of the diabetic state in antibody‐treated obese rats had an impact on the composition of ligature‐associated bacterial profiles in T2D, which was returned towards that of the lean animals.

In the present study, treatment with anti‐TNF‐α was associated with a bacterial profile different from that of the OPD group, which is interesting, since atypical infections have been reported in humans receiving anti‐TNF‐α (ETN) treatment. (Ellerin, Rubin, & Weinblatt, 2003). Furthermore, T2D has been reported to associate with an altered microbiological profile in the gut of humans, and this change has been proposed essential for the development of T2D (Qin et al., 2012). In line, in an animal study of female ZOR, the glycemic status was demonstrated to have an impact on the microbiota in the caecum with increased number of lactobacilli, which is parallel to the findings in the present study of the oral bacterial profile (Romo‐Vaquero et al., 2014). On another note, a study using HOMIM‐analysis showed that the bacterial profile is altered in Crohn's disease in humans (Docktor et al., 2012). Collectively, these findings illustrate that disease‐associated bacterial profiles may have the potential as future biomarkers for identification of chronic diseases like T2D.

In conclusion, the present study demonstrates that the composition of ligature‐associated bacterial profiles in a rat model is influenced by the diabetic state of the animal. Furthermore, treatment with anti‐TNF‐α or anti‐RAGE had an impact on both the diabetic state of the animals and ligature‐associated bacterial profiles. Future studies could be performed using next‐generation sequencing technologies addressing the specific microbiota associated with health and disease in diabetic rats.

## FUNDING INFORMATION

The study was supported by the Danish Medical Research Council, Hørslev‐Fonden, The Simon Spies Foundation, The Danish Dental Association, The Danish Diabetes Association, and the Danish Diabetes Academy supported by the Novo Nordisk Foundation.

## CONFLICT OF INTEREST

The authors declare that there are no conflicts of interest in this study.

## Supporting information

S1: Complete list of probes present on the HOMIM microarray.

S2: List of probes identified in subgingival ligatures in rats.
